# Shifts in composition and function of bacterial communities reveal the effect of small barriers on nitrous oxide and methane accumulation in fragmented rivers

**DOI:** 10.3389/fmicb.2023.1110025

**Published:** 2023-02-21

**Authors:** Chong-Yang Xing, Hang Li, Qi Li, Lun-Hui Lu, Zhe Li

**Affiliations:** ^1^Key Laboratory of Reservoir Aquatic Environment, Chongqing Institutes of Green and Intelligent Technology, Chinese Academy of Sciences, Chongqing, China; ^2^Chongqing School, University of Chinese Academy of Sciences, Chongqing, China

**Keywords:** river fragmentation, greenhouse gases, microbial community, habitats, low barriers

## Abstract

Rivers are often blocked by barriers to form different habitats, but it is not clear whether this change will affect the accumulation of N_2_O and CH_4_ in rivers. Here, low barriers (less than 2 m, LB) increased N_2_O concentration by 1.13 times and CH_4_ decreased by 0.118 times, while high barriers (higher than 2 m, less than 5 m high, HB) increased N_2_O concentration by 1.19 times and CH_4_ by 2.76 times. Co-occurrence network analysis indicated LB and HB can promote the enrichment of *Cyanobium* and *Chloroflexi*, further limiting complete denitrification and increasing N_2_O accumulation. The LB promotes methanotrophs (*Methylocystis, Methylophilus*, and *Methylotenera*) to compete with denitrifiers (*Pseudomonas*) in water, and reduce CH_4_ accumulation. While the HB can promote the methanotrophs to compete with nitrifiers (*Nitrosospira*) in sediment, thus reducing the consumption of CH_4_. LB and HB reduce river velocity, increase water depth, and reduce dissolved oxygen (DO), leading to enrichment of *nirS*-type denitrifiers and the increase of N_2_O concentration in water. Moreover, the HB reduces DO concentration and *pmoA* gene abundance in water, which can increase the accumulation of CH_4_. In light of the changes in the microbial community and variation in N_2_O and CH_4_ accumulation, the impact of fragmented rivers on global greenhouse gas emissions merits further study.

## Highlights

- Small barriers can promote the accumulation of N_2_O and CH_4_ in fragmented river.
- Enrichment of *Cyanobium*, *Chloroflexi*, *nirS*-denitrifiers increases N_2_O accumulation.
- Small barriers promote the methanotrophs to compete with *Nitrosospira* in sediment.
- Small barriers reduces DO concentration and *pmoA* gene abundance in water.

## 1. Introduction

Natural rivers are typically characterized by their free-flowing, and they are often fragmented by barriers to free flow (Grill et al., 2019). Artificial interception (barriers) causes fragmentation and blockage of urban rivers (Grill et al., 2019), resulting in the formation of different habitats of rivers (Carpenter et al., 2011). There are more than 1 million barriers cause of rivers fragmentation (Belletti et al., 2020). Barriers higher than 15 m are rare, 68% had less than 2 m height and 91% had less than 5 m height (Belletti et al., 2020), leading to a major fragmentation caused by small barriers (Jones et al., 2019). However, the environmental effects of small barriers on rivers are not clear, especially whether it affects the accumulation of greenhouse gases in rivers.

There is growing evidence that urban river networks may be hot spots for greenhouse gas (N_2_O, CH_4_) emissions (Zhang W. et al., 2021). Inland waters are significant emitters of N_2_O (Gongqin et al., 2021), and the total estimated N_2_O from rivers is approximately 1.05 Tg N-N_2_O Yr^−1^ (global total emissions are 17.7 Tg N-N_2_O Yr^−1^; Frostegard et al., 2022). CH_4_ is generally considered to be a more important greenhouse gas than N_2_O (Stein, 2020; Neubauer, 2021). An increasing number of studies have shown that river interception significantly damages river continuity and flood pulsation (Wang et al., 2010), changed the fluxes and ecological dynamics of water and nutrients (Deemer et al., 2016) and affected the migration and transformation process of nitrogen and carbon.

The production of N_2_O is strongly affected by microorganisms and mainly involves the oxidation and reduction of active nitrogen (ammonia, nitrate, and nitrite; Babbin and Ward, 2013). N_2_O in rivers is thought to be formed in the bottom sediment, where a variety of microbes produce a large amount of N_2_O (Beaulieu et al., 2011). Incomplete denitrification is the main cause of N_2_O production in rivers (Clough et al., 2007; Babbin and Ward, 2013; Lansdown et al., 2015). Variation in river hydrological conditions affects undercurrent exchange, which in turn affects river water quality (such as the reactive nitrogen load) and ultimately N_2_O production (Marzadri et al., 2014). Given the complexity of N_2_O generation, N_2_O accumulation in different habitats of fragmented rivers is still uncertain, especially the effects on the structure and function of N_2_O accumulation-related microbial communities.

Biomethane sinks are composed mainly of methanophile microorganisms that use methane monooxygenase to oxidize CH_4_ to methanol using oxygen (Kallistova et al., 2017), which is then oxidized to formaldehyde, formic acid and carbon dioxide by a series of enzymes. It’s estimated that microbial oxidation can remove about 5% of CH_4_ emissions into the atmosphere each year (Stein, 2020). Current studies suggest that O_2_ is the factor that strongly affects microbial metabolism and CH_4_ emissions in river ecosystems (Kallistova et al., 2017). The aggregation of multiple microbial processes in river aerobic/anoxic zones suggests that these zones are highly dynamic in controlling CH_4_ fluxes and may be the most important region for CH_4_ mitigation. The riverine barriers slow the flow of fragmented rivers and increase the depth of water, which may lead to a decrease in the concentration of dissolved oxygen (DO) in the water. Changes in the DO concentration of fragmented rivers may lead to changes in microbial activity related to CH_4_ oxidation and thus affect CH_4_ accumulation in the rivers. Therefore, it is not clear if the effect of barriers over DO may lead to changes in CH_4_ oxidation, thus affecting CH_4_ accumulation.

In this study, Liangtan river (China) was studied as an example of fragmented river, where four habitats were delimited according to the height of the barriers between them. High-throughput sequencing was used to analyze the water and sediment microbial communities to explore how the barriers changed the microbial network structure in the four habitats. Quantitative PCR was used to analyze the changes in gene abundance of functional enzymes, further revealing the metabolic process of N_2_O and CH_4_ accumulation in different habitats of fragmented rivers.

## 2. Materials and methods

### 2.1. Site description and sampling

**Figure 1:**
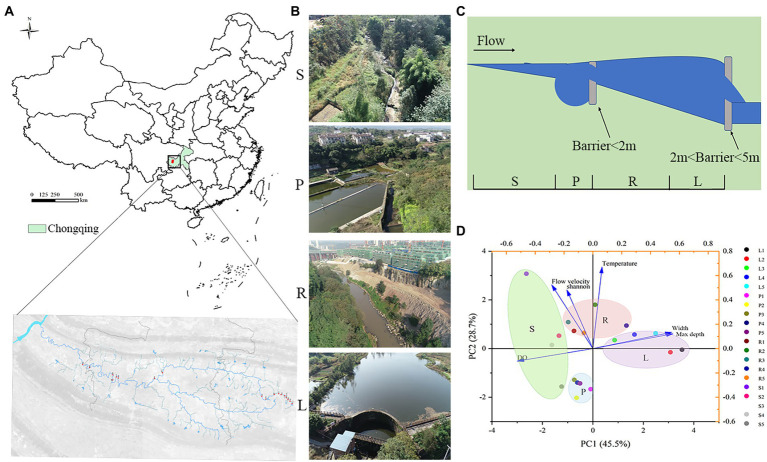
According to the height of artificial barriers above the river (lower than 2 m or lower than 5 m), river channels are divided into four habitats. For LB (barriers less than 2 m high), the matrix habitat formed is called ‘pond’ (P), while the patch habitat is called ‘stream’(S). For HB (barriers less than 5 m high), matrix habitat formed by barriers is called ‘lake’ (L), while patch habitat is called ‘river’ (R). **(A)** Sampling locations of 20 sites on the Liangtan River. **(B)** Photographs of the sampling sites in this study. **(C)** Diagram of river channels division. **(D)** 20 water samples from four habitats were analyzed by Euclidean distance-based principal component analysis (PCA).

The Liangtan River (29°26′–29°52′ N, 106°18′–106°24′ E) is a first-class tributary of the lower right bank of the Jialing River (Figure 1A). In a preliminary investigation, we found more than 15 barriers that led to fragmentation of the river. According to the barrier height, the Liangtan River can be divided into four habitats: pond (P) and stream (S) for low barriers (less than 2 m high, LB) and lake (L) and river (R) for high barriers (between 2 m and 5 m, HB; Figure 1; Fuller et al., 2015; Belletti et al., 2020).

Samples were collected from the four habitats of the Liangtan River (December 2020 and August 2021). Sediment samples were obtained at a depth of 10 cm below the interface sediment: water, whereas water samples were obtained from a total of 20 sampling sites in the river channels. Sediments were immediately placed in sterile bags and sealed, placed on ice bags, shipped back to the laboratory within 12 h, and stored in a −80°C freezer. The water samples were divided into two parts. The first part was supplemented with HgCl_2_ (20 μg/mL) for subsequent determination of water quality indicators. The other part was returned to the laboratory within 12 h and filtered through a 0.22-μm glass fiber membrane to remove trapped microorganisms for subsequent high-throughput sequencing. Characteristics of the 20 sites studied (5 sites per habitat) in the Liangtan River are shown in Supplementary Table S1.

### 2.2. Chemical analysis of water quality parameters

Electrical conductivity (EC), water temperature (T), and DO were determined by a YSI^®^ ProODO DO meter, and the pH was determined by a YSI^®^ 63 pH meter. Total organic carbon (TOC) was determined by a Shimazu^®^ TOC-VWP analyzer (Shimadzu^®^, Japan). Chlorophyll *a* (Chl *a*) was extracted by ethanol and determined by spectrophotometry (Ortega et al., 2019). Total nitrogen (TN) was measured by the potassium persulfate oxidation method (Zhang L. Y. et al., 2021). Total phosphorus (TP) was extracted by HClO_4_-H_2_SO_4_ and measured by the molybdenum blue method. Nitrite nitrogen (NO_2_^−^-N) was determined by N-(1-)-ethylenediamine spectrophotometry. Nitrate nitrogen (NO_3_^−^-N) was measured by potassium peroxodisulfate solution spectrophotometric methods. Ammonium nitrogen (NH_4_^+^-N) was measured by spectrophotometry with a Nessler reagent (Ministry of Environmental Protection, 2012).

### 2.3. High-throughput sequencing analysis

Total DNA was extracted from 0.5-g sediment samples using a Rapid Soil DNA Isolation Kit. The concentration of the extracted DNA was then quantified using an ND-2000C spectrophotometer (Nanodrop, Thermo Scientific, United States), and the extracted DNA was used for high-throughput sequencing. The V3-V4 hypervariable region of the 16S rRNA gene was amplified with 338F/806R universal primers (Supplementary Table S2), and each sample was identified. Specific PCR amplification conditions of the 16S rRNA gene were based on those described in previous studies (Junfeng et al., 2015). The resulting PCR products were then sequenced on an Illumina HiSeq 2000 platform. Data processing and statistical analysis were carried out (see the attachment for specific methods). The raw data of Illumina Miseq sequencing were submitted to the NCBI under the BioProject accession number PRJNA904922.

### 2.4. Measurement of N_2_O and CH_4_ concentrations in water

The concentrations of N_2_O and CH_4_ in water were measured using the static headspace method. In this study, the traditional method (Donis et al., 2017) was improved. Firstly, a syringe is used to extract 200 mL water sample from the water sampler. The sample should be extracted slowly during the collection process to avoid bubbles. Extract 100 mL nitrogen from an air bag containing high purity nitrogen (99.999% purity) (500 mL air bag) to form an air chamber above the syringe (the same syringe used to collect water sample). Hold the syringe and shake it up and down for 3 min to achieve a balance between the gas–liquid phase. Push the gas in the syringe into the prepared vacuum air bag (300 mL air bag) for preservation. Record the temperature of the water after the shake. The preserved headspace samples were returned to the laboratory and the concentration of N_2_O and CH_4_ was determined by gas chromatography.

### 2.5. Quantitative PCR

16S rRNA and the abundance of functional genes of denitrifying bacteria were detected by qPCR using the Majorbio Cloud Platform (Shanghai). The functional genes were mainly divided into nitrite reductase (*nir*), NO reductase (*nor*), N_2_O reductase (*nos*), and particulate methane monooxygenase (*pmo*). Three repeats of qPCR were performed on each sample using an ABI 7500 sequence detection system with the SYBR method (Applied Biosystems, Canada). Primer sequences are listed in Supplementary Table S2. qPCR-specific tests were performed using a melting profile and gel electrophoresis to reduce the possibility of overestimating gene abundances. In addition, each qPCR reaction consisted of negative control, in which no DNA template was added. The abundance of functional genes was converted to the number of copies of functional genes per gram of dry sediment or per milliliter of water. The efficiency was assumed to be 100% for DNA extracted from sediment or water samples (Zheng et al., 2019).

### 2.6. Statistical analyses

One-way ANOVA and Tukey’s *post hoc* tests were used to evaluate the differences in microbial diversity and abundance, structure, and environmental factors among habitat types. The above statistical analyses were conducted using PASW Statistics 18 software (IBM SPSS Inc., Chicago, United States). The molecular ecological networks of habitats S, P, R, and L were constructed to analyze the microbial community structure. Linear discriminant analysis (LDA) was used to estimate biomarkers of the four habitats based on analysis software (LEfSe). Based on Origin, a heatmap was drawn to analyze the microbial abundance related to the production of N_2_O and CH_4_ in the four habitats. To further elucidate the direct and indirect effects of environmental factors and microbial communities on N_2_O concentrations and CH_4_ concentrations, we conducted path analyses using the maximum likelihood estimation method. Gephi version 0.8.2 was used for network visualization and modular analysis. According to Banerjee et al. (2018), keystone species were identified using the following thresholds: genera with high mean degree (>30) and low betweenness centrality (<700) in the network. Path coefficients, *R*^2^, direct and indirect effects, and model fit parameters were calculated using R studio.

## 3. Results

### 3.1. Differences in diversity and community composition of water and sediment bacteria in four habitats

Samples were taken from five randomly selected points in each habitat (Figures 1A–C). Through principal component cluster analysis of physical and chemical indexes of four habitats, it was found that the four habitats could be distinguished well, which indicated that it was appropriate to divide the different habitats of Liangtan River (Figure1D). The abundance and composition at the phylum level of microbial communities from water and sediment was analyzed for each habitat (S, P, R, and L; Supplementary Figure S1). A total of 14,481,930 high-quality bacterial 16S rRNA gene sequences and 30,863 operational taxonomic units (OTUs) were obtained by high-throughput sequencing.

**Figure 2:**
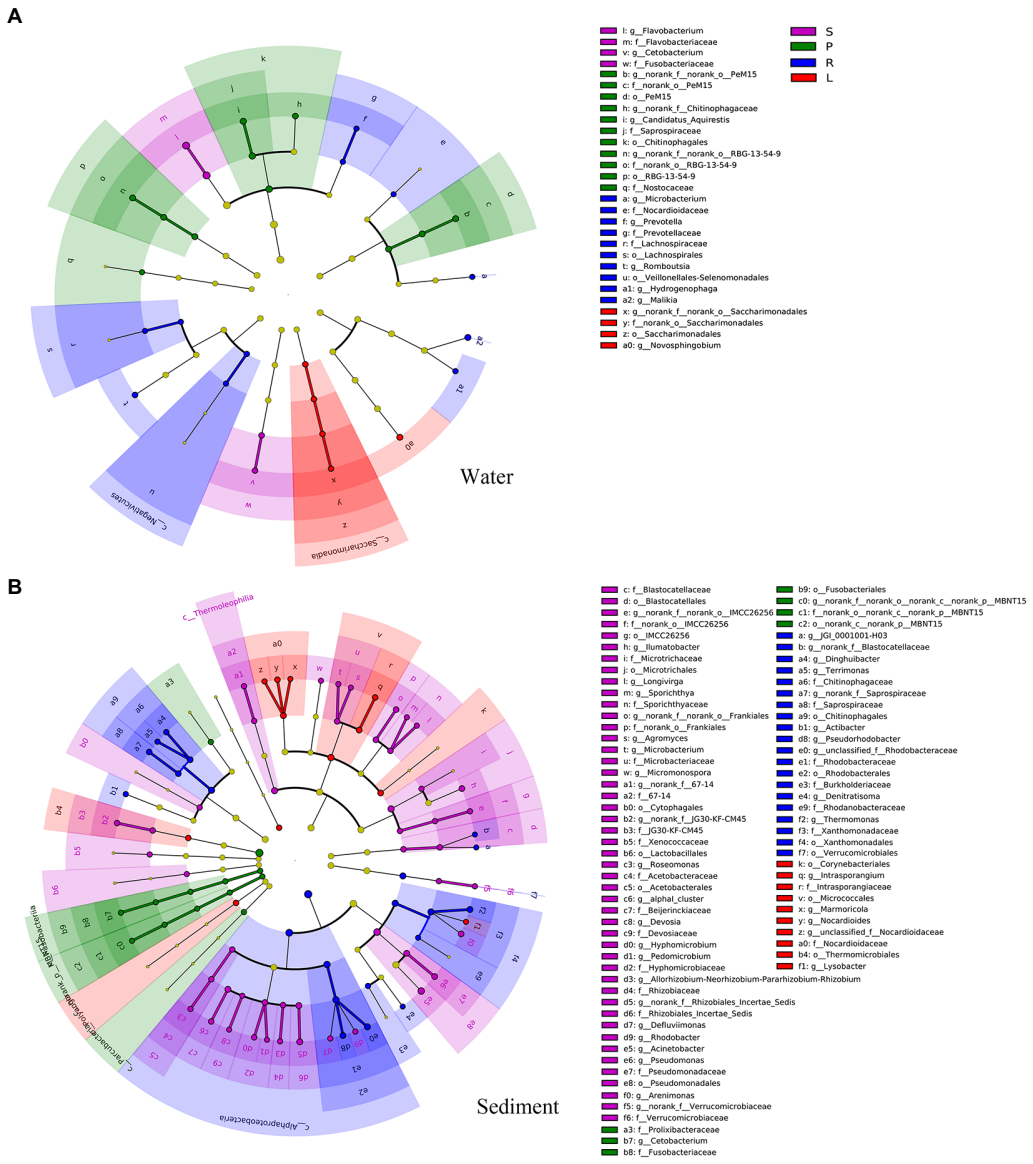
The abundant biomarkers in water **(A)** and sediment **(B)** obtained for four habitats. All detected taxa, with a relative abundance of >0.5% in at least one sample, were assigned to phyla (outermost), classes, orders, families, and genera (innermost) and were used to determine the taxa or clades most likely to explain differences between the four habitats.

The relative abundances of the phyla *Proteobacteria*, *Bacteroidota*, and *Cyanobacteria* were higher in water (17.6–76.5%, 2.8–57.9%, and 0.2–41.4%, respectively) than in sediment (7.0–45.8%, 1.3–9.2%, and 0.2–4.2%, respectively). In contrast, the relative abundances of *Chloroflexi*, *Firmicutes*, *Acidobacteriota*, *Verrucomicrobiota*, and *Desulfobacterota* were higher in sediment (4.3–35.3%, 2.0–31.1%, 2.2–14.8%, 1.5–10.0%, and 0.1–9.0%, respectively) than in water (0.05–6.5%, 0.7–13.5%, 0.03–0.9%, 0.1–4.5%, and 0.06–1.0%, respectively, Supplementary Figure S2). Most of these biomarkers were abundant in the sediment of S and R. There were 31 and 102 biomarkers (*p* < 0.05, LDA > 2.0) in water and sediment, respectively (Figure 2). The classes *Acetobacteraceae*, *Blastocatellales*, *Microtrichales*, and *Pseudomonadales* were the main biomarkers in sediment samples from S. Species belonging to *Rhodobacterales*, *Xanthomonadales*, and *Chitinophagales* dominated in sediment samples from R. *Flavobacteriaceae* and *Fusobacteriaceae* species were most abundant in water samples from S. Species affiliated with *Veillonellales*, *Prevotellaceae*, *Nocardioidaceae*, and *Lachnospirales* were abundant in water samples from R (Figure 2). The number of biomarkers was lower in L and P. In L, the classes *Ncardioidaceae* and *Micrococcales* were the main biomarkers in sediment, and *Saccharimonadales* was dominant in water. In P, the classes *Norank*, *Fusobacteriales*, and *Prolixibacteraceae* were the main biomarkers in sediment, whereas *RBG-13-54-9*, *Chitinophagales*, and *PeM15* were dominant in water.

### 3.2. Water and sediment bacteria of four habitats exhibited contrasting ecological network patterns

**Figure 3:**
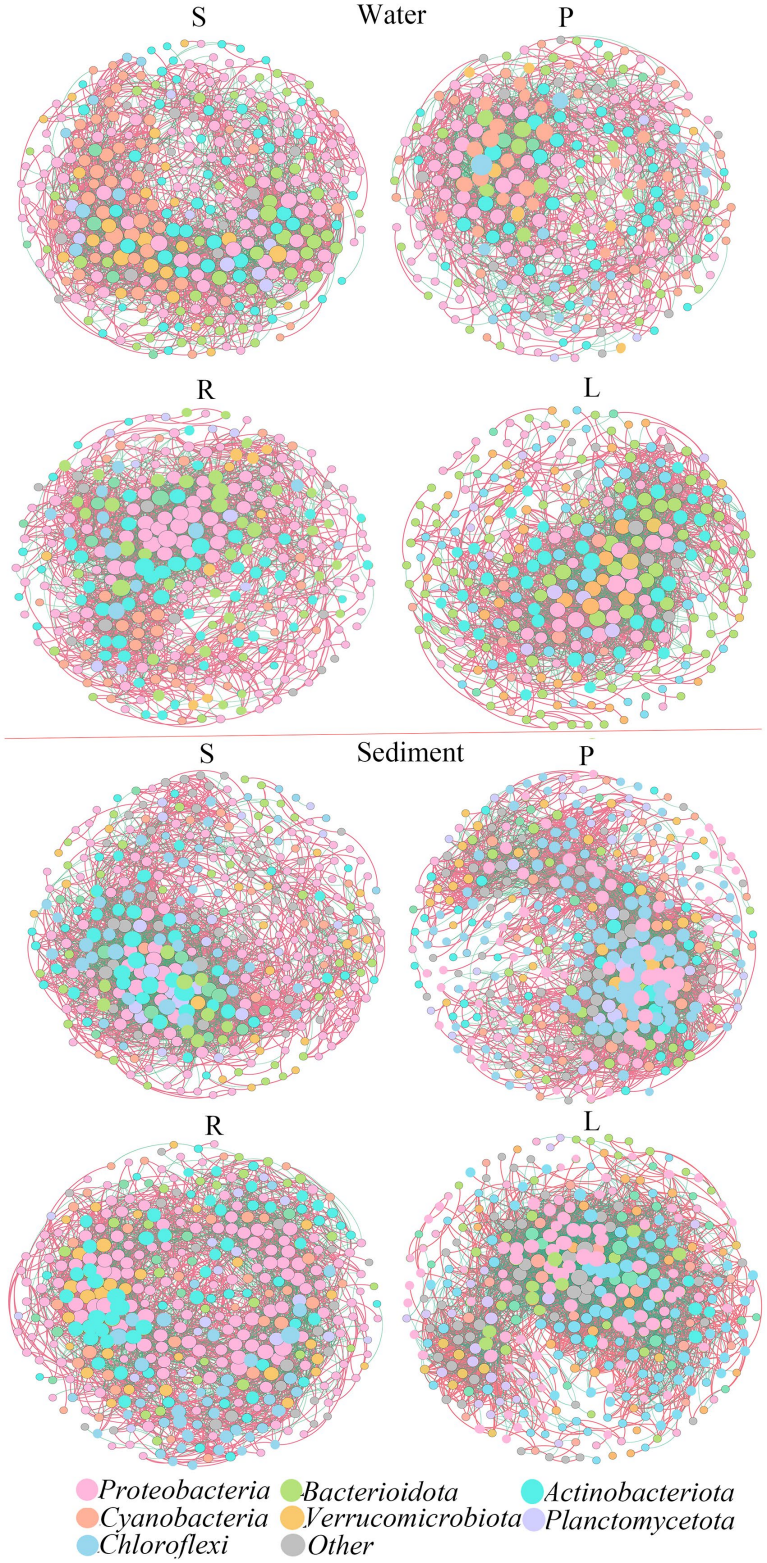
Co-occurrence networks and their topological properties of different bacterial communities in the water and sediment.

The topological structures of the bacterial communities at the OTU level are shown in Figure 3. Regarding the sediment samples, the percentage of *Chloroflexi* nodes increased in P compared with that in S, while the node ratio in *Proteobacteria* and *Actinobacteriota* decreased. Similarly, the percentage of *Chloroflexi* nodes increased in P compared with that in S, while the node ratio in *Proteobacteria* and *Actinobacteriota* decreased.

Then we further analyzed the variation of ecological network parameters in the four habitats. The two networks were defined according to the same threshold, and the corresponding statistical results are shown in Supplementary Table S3. In water, the modularity of the S bacterial network (1.734) was lower than that of the P bacterial network (3.147). In S, the resulting networks consisted of 334 nodes linked by 2,809 edges, which consisted of 1,816 positive correlations and 993 negative correlations. In P, the resulting networks consisted of 287 nodes linked by 2,105 edges, which consisted of 1,210 positive correlations and 895 negative correlations. Compared with R, the modularity of L was lower. In R, the resulting networks consisted of 320 nodes linked by 2,812 edges, which consisted of 1,759 positive correlations and 1,042 negative correlations. In L, the resulting networks consisted of 300 nodes linked by 2,816 edges, which consisted of 1,764 positive correlations and 1,052 negative correlations. The presence of barriers will reduce the number of nodes, and positive or negative correlations in the microbial network, leading to the estrangement of microbial connections in the water.

In sediments, the modularity of S was higher than that of P, and that of L was higher than that of R. In S, the network consisted of 370 nodes and 3,756 edges. The network showed 2,293 positive correlations, which was higher than the negative correlations (1,463 green lines). In P, the network consisted of 388 nodes and 4,789 edges. The network showed 2,967 positive correlations and 1,822 negative correlations. In R, the network consisted of 409 nodes and 4,128 edges. The network showed 2,730 positive correlations and 1,428 negative correlations. In L, the network consisted of 373 nodes and 4,733 edges. The network showed 2,735 positive correlations and 1,999 negative correlations. This suggested that the presence of obstacles may lead to closer connections of microbial networks in sediments.

### 3.3. Effects of microbial composition in four habitats on CH_4_ and N_2_O accumulation

**Figure 4:**
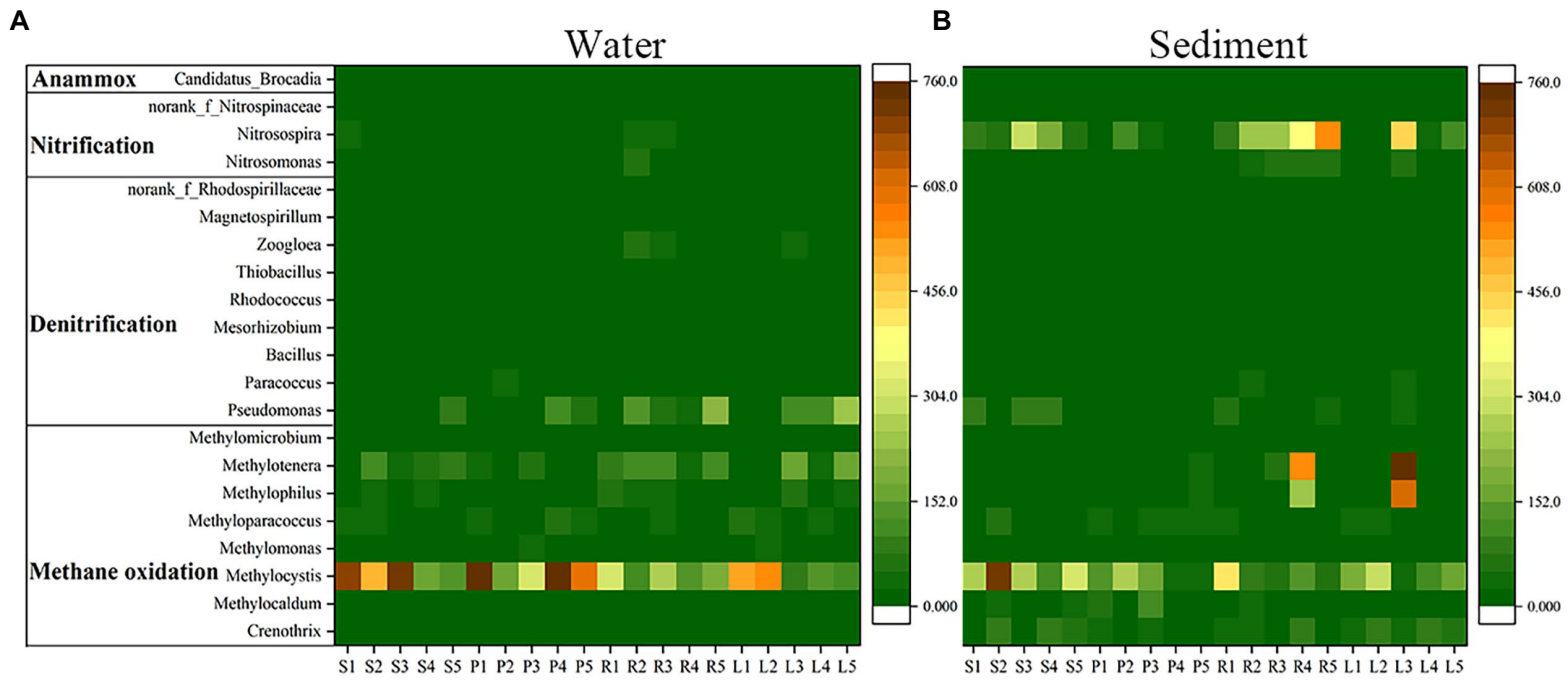
Heatmap of anammox bacteria, nitrifiers, denitrifiers and methanotrophs (average abundance in all samples) of water and sediment in four habitats.

In water, the abundances of anammox bacteria and nitrifiers were low, and denitrifiers (*Pseudomonas*) and methanotrophs (*Methylocystis*) were more abundant (Figure 4A). The denitrifying bacterial abundance of P was higher than that of S, and that of L was higher than that of R. The methanotrophs abundance of S was higher than that of P, and that of L was higher than that of R. In the sediment, the abundances of anammox bacteria and denitrifiers were low, the nitrifiers (*Nitrosospira*) and methanotrophs (*Methylophilus*, *Methylocystis*, *Methylocystis*) were more abundant (Figure 4B). The nitrifying bacterial abundance of S was higher than that of P, and that of R was higher than that of L. The methanotrophs abundance of S was higher than that of P, and that of L was higher than that of R.

**Figure 5:**
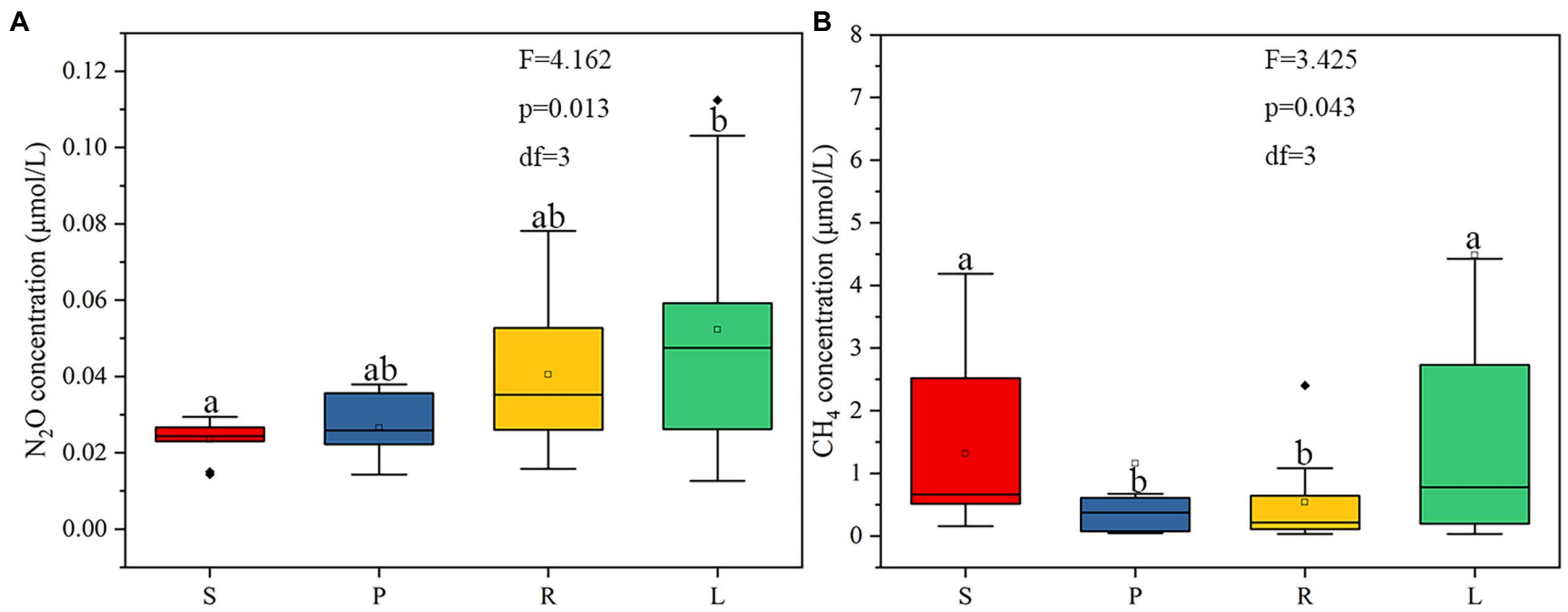
The concentration of N_2_O **(A)** and CH_4_ **(B)** in four habitats.

The concentrations of dissolved N_2_O in S, P, R, and L were 0.014–0.029, 0.014–0.037, 0.019–0.078, and 0.012–0.11 μmol/L, respectively (Figure 5A). Compared with S, the concentration of N_2_O in P showed an increasing trend. The concentration of N_2_O in L was higher than that in R. LB and HB can increase the N_2_O concentration in rivers by 1.13 and 1.29 times, respectively. The concentrations of dissolved CH_4_ in S, P, R, and L were 0.30–4.18, 0.04–0.67, 0.03–2.4, and 0.03–34.0 μmol/L, respectively (Figure 5B). The CH_4_ concentration in S was higher than that in P, and that in L was higher than that in R, it can be inferred that that LB reduces CH_4_ accumulation and HB increase it. LB reduced the CH_4_ concentration by 0.118 times, while HB increased it by 2.76 times.

**Figure 6:**
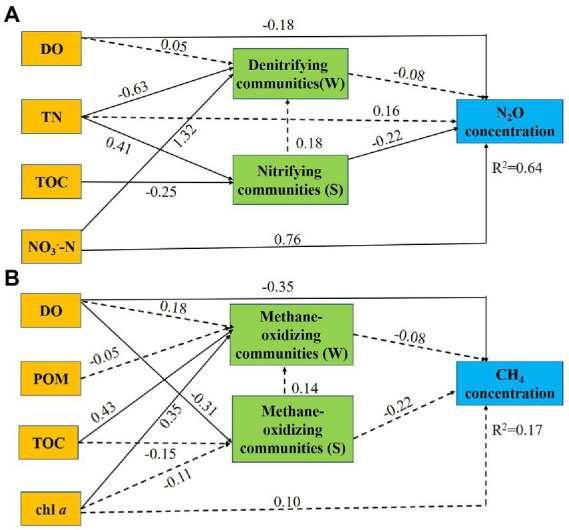
Path diagrams estimating the direct and indirect effects of environmental factors and microbial communities on N_2_O concentrations **(A)** and CH_4_ concentrations **(B)**. Solid lines demonstrate significant effects (*p* < 0.05), and dashed lines indicate insignificant effects. Numbers adjacent to the arrows are standardized path coefficients. Single headed arrows refer to unidirectional causal relationships. Denitrifying community (W): abundance of denitrifiers in water. Nitrifying community (S): abundance of nitrifiers in sediment. Methane-oxidizing community (W): abundance of methanotrophs in water. Methane-oxidizing community (S): abundance of methanotrophs in sediment.

Next, we conducted path analyses to further elucidate the direct and indirect effects of environmental factors and microbial communities on N_2_O concentrations and CH_4_ concentrations. DO and NO_3_^−^-N could regulate the concentration of N_2_O in water both directly and indirectly (Figure 6A). The regulation of DO to N_2_O is reverse, while that of NO_3_^−^-N is positive. DO (*β* = −0.18, standardized coefficient) directly impacted the concentration of N_2_O. NO_3_^−^-N was the most significant parameter (*β* = 0.76, standardized coefficient) influencing the concentration of N_2_O. NO_3_^−^-N indirectly through water denitrifying communities (*β* = 1.32, standardized coefficient) impacted the concentration of N_2_O. Furthermore, TN indirectly through sediment nitrifying bacterial community (*β* = 0.41, standardized coefficient) impacted the concentration of N_2_O. Moreover, DO directly (*β* = −0.35, standardized coefficient), and indirectly through soil pH (*β* = −0.31, standardized coefficient), impacted the concentration of CH_4_ (Figure 6B).

### 3.4. Relative contribution of genes (*nirS*, *nirK*, *nosZ*, and *pmoA*) in four habitats to N_2_O and CH_4_ accumulation

**Figure 7:**
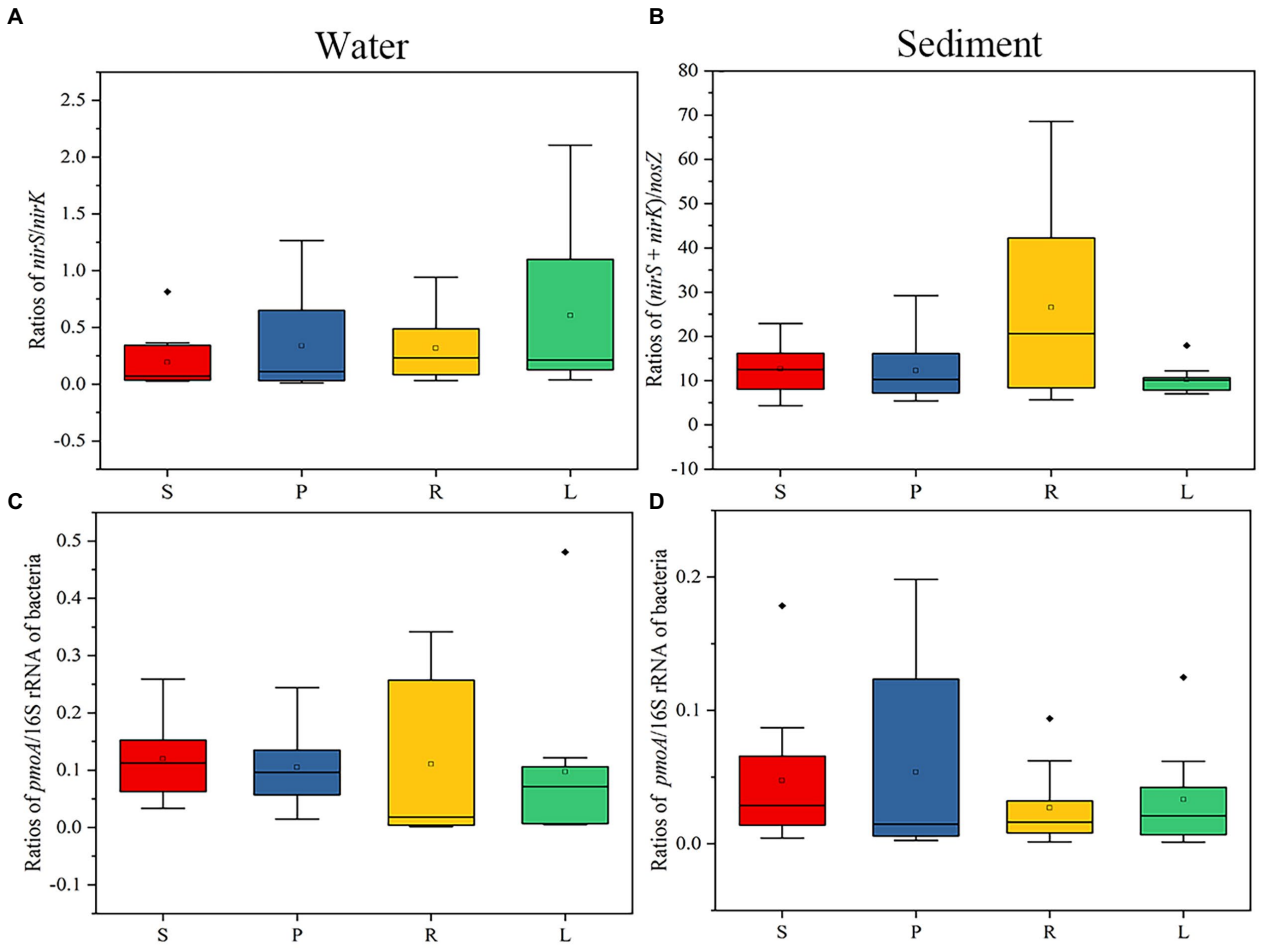
Gene ratios of functional enzymes in water and sediment of four habitats. **(A)** *nirS/nirK* copy number ratios in water. **(B)** (*nirS + nirK*)/*nosZ* copy number ratios in sediment. **(C)** *pmoA*/16S rRNA copy number ratios in water. **(D)** *pmoA*/16S rRNA copy number ratios in sediment. The bacteria represent the abundance of the total bacterial 16S rRNA gene.

As can be seen from the above analysis, the LB and HB promote the enrichment of denitrifiers in the P and L habitat. To further verify our hypothesis, the abundances of functional enzyme genes of denitrifiers of water were analyzed in four habitats. The abundances of *nirS*, *nirK*, and *nosZ* in water and sediment were counted (Supplementary Tables S6–S9). To estimate the abundance contribution of different genes in the denitrification pathway to N_2_O accumulation, the *nisS/nirK and* (*nisS + nirK*)/*nosZ* copy number ratios were analyzed (Figure 7). The *nirS*/*nirK* of P and L was higher than that of S and R, suggesting that the LB and HB promote the enrichment of *nirS*-type denitrifiers, and then affect the accumulation of N_2_O. In sediments (*nirS* + *nirK*)/nosZ copy number ratios were decreased in P and L compared with S and R, suggesting that the LB and HB promoted the *nosZ* enrichment in sediments.

Then, the abundances of *pmoA* in water and sediment were counted (Supplementary Tables S6–S9). To estimate the contribution of functional genes to the abundance of CH_4_ accumulation in CH_4_ oxidation, we analyzed the *pmoA*/16S rRNA copy number ratio in water and sediment of four habitats (Figure 7). *pmoA* is a key enzyme gene for CH_4_ oxidation, which reduce CH_4_ accumulation (Lieberman and Rosenzweig, 2004; Vrieze and Verstraete, 2016). In water, the *pmoA*/16S rRNA copy number ratio of R was the highest, while in sediment, the *pmoA*/16S rRNA copy number ratio of P was the highest. These results further suggest that the presence of small barriers affects CH_4_ accumulation by altering the activities of enzymes involved in methane oxidation.

## 4. Discussion

### 4.1. Small barriers affect N_2_O and CH_4_ accumulation

LB and HB lead to lower flow rates and increased water depth in fragmented rivers, which change environmental variables and affect N_2_O and CH_4_ accumulation. The small barrier leads to an increase in the concentration of NO_3_^−^-N (Supplementary Tables S4, S5), which promotes the accumulation of N_2_O in P and L. Both LB and HB reduce river velocity, leading to an increase in the water bed contact area per unit of water volume, and an increase in the diffusion efficiency of NO_3_^−^-N at the river-sediment interface (Mulholland et al., 2008). This is consistent with previous work showing that nitrate concentrations strongly affect the production of N_2_O (Meyer et al., 2008). With few exceptions, the nitrate concentration in sediment and surface water is positively correlated with the production of N_2_O (Quick et al., 2019). The positive correlation between N_2_O and NO_3_^−^-N might stem from incomplete denitrification (Beaulieu et al., 2011; Abed et al., 2013; Jung et al., 2019). LB and HB promoted the enrichment of denitrifiers in the water of P and L respectively, which may lead to an increase in incomplete denitrification under unit nitrogen load (Mulholland et al., 2008).

Moreover, in most rivers, the overlying water above the surface of the sediment is anoxic and carries dissolved oxygen that continuously seeps into the sediment (Xia et al., 2018). The depth of dissolved oxygen penetration into sediment is affected by sediment roughness, porosity, and connectivity (Xia et al., 2018). LB and HB lead to increased water depth and decreased DO concentration in the river (Supplementary Figure S3), which is conducive to incomplete denitrification.

In addition, the higher abundance of nitrifiers (*Nitrosospira*) in the lotic habitats (S and R) suggest that the LB and HB would inhibit the growth of nitrifiers. The main reason is that the barriers reduce the DO in the river, thus inhibiting the growth of nitrifiers (aerobic bacteria). At present, there are still many uncertainties about the relationship between nitrifiers and N_2_O accumulation (Beaulieu et al., 2010). According to our results, the decrease of nitrifiers promotes N_2_O accumulation to a certain extent in river surface sediments.

According to the CH_4_ concentration in four habitats, the CH_4_ concentration in P was lower than in S. The main reason was that the abundance of methanotrophs in P was higher than that in S, resulting in a large amount of CH_4_ consumption. It can be seen that the LB reduces CH_4_ accumulation. Compared with R, the CH_4_ concentration in L was higher. The potential reason was that the concentration of DO in L was reduced, which was conducive to the generation of CH_4_ under anaerobic conditions. It can be seen that the HB increases the accumulation of CH_4_, these results were consistent with other literature reports (He et al., 2021).

### 4.2. Microbial network and functions of water and sediment in the four habitats

Denitrifiers and methanotrophs were abundant in the four habitats and significantly affected N_2_O production and CH_4_ oxidation, respectively. Hence, a co-occurrence network analysis was conducted to identify the cooperative and competitive relationships among denitrifiers, methanotrophs, and other microorganisms. In networks of P, *Proteobacteria* was still the first dominant phylum, and the second dominant phylum was *Cyanobacteria*. *Cyanobium_PCC-6307* is a genus with high content of *Cyanobacteria* phylum. It has been reported that the abundance of denitrifiers has a competitive relationship with non-diazotrophic *Cyanobium* sp. (Song et al., 2022), so it is further speculated that LB increases the abundance of *Cyanobium*, further limits complete denitrification and increases N_2_O accumulation. Interestingly, denitrifiers (*Pseudomonas*) and methanotrophs (*Methylocystis*, *Methylophilus, Methylotenera*), were dominant nodes in water. Previous studies have also reported low N_2_O and high CH_4_ fluxes in rivers, which they suggest are due to competition (Deemer et al., 2016). The potential cause is the production of a copper chelator (methanobactin) by methanotrophs during the CH_4_ cycle (Spirito et al., 2016), which has been shown to effectively compete for copper from denitrifiers, thereby increasing N_2_O production while reducing CH_4_ emissions (Chang et al., 2018). Similarly, the availability of copper is particularly important for *nosZ* expression and activity, since copper is required for the active site of the enzyme (Gaimster et al., 2018). *nosZ* will compete with methanotrophs for copper ions, thus reducing N_2_O production and increasing CH_4_ accumulation. This is consistent with our results that the ratio of *nosZ* and concentration of CH_4_ in S and L is higher than that of P and R (Supplementary Figure S4).

In sediments, the *Chloroflexi* was the dominant bacteria in P and L habitats, it was speculated that the accumulation of *Chloroflexi* may promote the accumulation of N_2_O. It has been reported that bacteria attached to *Chloroflexi* may be highly active protein degradation, breaking down the extracellular peptides bound to the extracellular polymer matrix and simultaneously breathing nitrate to produce nitrite (Lawson et al., 2017). The increase of nitrite can promote denitrification (Quick et al., 2019), and thus increase the accumulation of N_2_O, which further proves that the barriers can increase the accumulation of N_2_O. Unlike in water, nitrifiers (*Nitrosospira*) and methanotrophs (*Methylocystis*, *Methylophilus*, *Methylotenera*), were dominant nodes in sediments. It has been reported that nitrifiers can be inhibited by methanotrophs due to competition between nitrifiers and methanotrophs for available oxygen and inorganic nitrogen (Megmw and Knowles, 1987). As LB and HB reduce DO concentration, nitrifiers in P and L were more severely inhibited compared with S and R, resulting in the stronger activity of methanotrophs. On the other hand, ammoxidation microorganisms may consume CH_4_, thereby reducing the amount of CH_4_ oxidized by methanotrophs (Bodelier and Laanbroek, 2004), and thus competing with methanotrophs for CH_4_. Therefore, the decrease of CH_4_ content in habitats P and R was also related to the nitrifiers.

### 4.3. Accumulation patterns of N_2_O and CH_4_ in fragmented rivers

**Figure 8:**
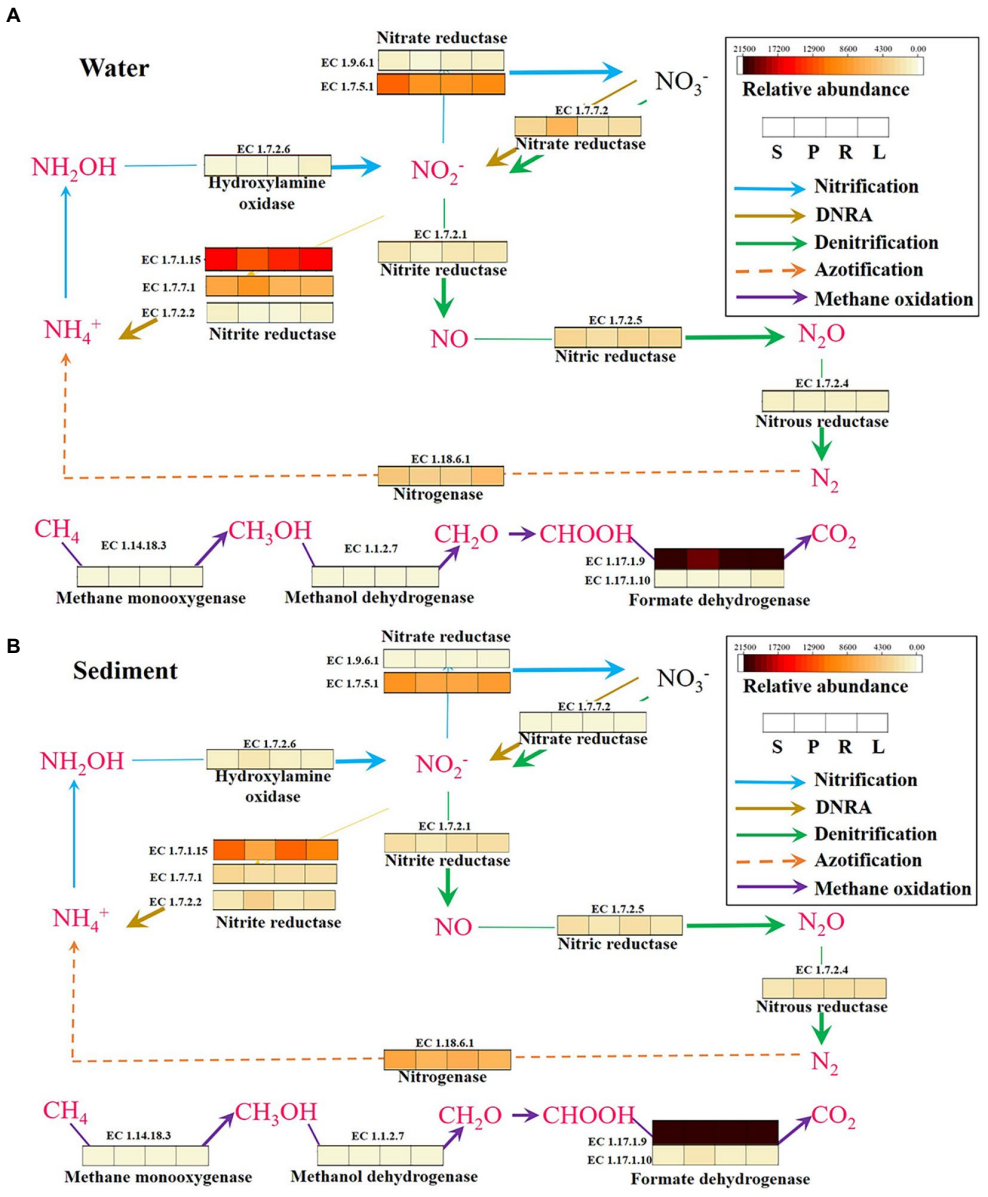
KEGG pathways and functional genes related to N_2_O and CH_4_ metabolism in the water **(A)** and sediment **(B)** of four habitats. DNRA, dissimilatory nitrate reduction to ammonium.

In order to further analyze the accumulation patterns of N_2_O and CH_4_ in fragmented rivers, the potential metabolic pathways of N_2_O and CH_4_ in different habitats were further analyzed. Compared with microbial functional groups, the metabolism between functional enzyme genes can better reveal the coupling relationship of element metabolic processes in environmental media. First, the potential N_2_O metabolism pathways of microorganisms in four habitats were analyzed. The analysis of the metabolic processes of nitrogenous microorganisms in four habitats revealed that the content of enzymes ([EC:1.7.7.2]) involved in nitrate transformation processes was higher in P compared with S in water (Figure 8), and this promoted the generation of N_2_O. This further proves that LB promotes the generation of N_2_O. The expression of enzyme genes involved in the dissimilatory nitrate reduction to ammonium (DNRA) process (Li et al., 2019) ([EC:1.7.1.15], [EC:1.7.7.1], [EC:1.7.2.2]) was higher in S than in P in sediment. The results indicated that more NO_2_-was converted into NH_4_^+^ in S, which reduced the possibility of N_2_O formation. The content of enzymes ([EC:1.7.2.6]) involved in nitrate transformation processes was higher in L compared with R in water and sediment, and indirectly promotes the generation of N_2_O. The expression of enzyme genes involved in the DNRA process ([EC:1.7.1.15]) was higher in R than in L in sediment. The results indicated that more NO_2_^−^ was converted into NH_4_^+^ in R, which reduced the possibility of N_2_O formation. Some studies have shown that the growth rate of a new class of non-denitrifying N_2_O reductants may be slow, but the isolated strains have great metabolic flexibility, enabling them to grow through DNRA (Sanford et al., 2012; Jones et al., 2014), which plays an important role in N_2_O reduction. It indicates that both LB and HB can promote the DNRA process, thus increasing the accumulation of N_2_O.

The analysis of the metabolic processes revealed that the content of enzymes (EC:1.17.1.10; Li et al., 2019) involved in CH_4_ oxidation processes was higher in P compared with S in sediment (Figure 8), and directly reduced the generation of CH_4_. It further shows that LB inhibits CH_4_ accumulation by promoting CH_4_ oxidation. The enzymes (EC:1.14.18.3; Zhang et al., 2020) involved in CH_4_ oxidation processes were higher in R compared with L in water and sediment, and directly reduced the accumulation of CH_4_. It further shows that HB increases CH_4_ accumulation by inhibiting CH_4_ oxidation. Some studies have speculated that aerobic CH_4_ oxidation coupled to denitrification process and anaerobic nitrite-dependent CH_4_ oxidation processes in river sediments (Zhang et al., 2020). It was speculated that the barriers may affect these two processes and thus the accumulation of N_2_O and CH_4_.

**Figure 9:**
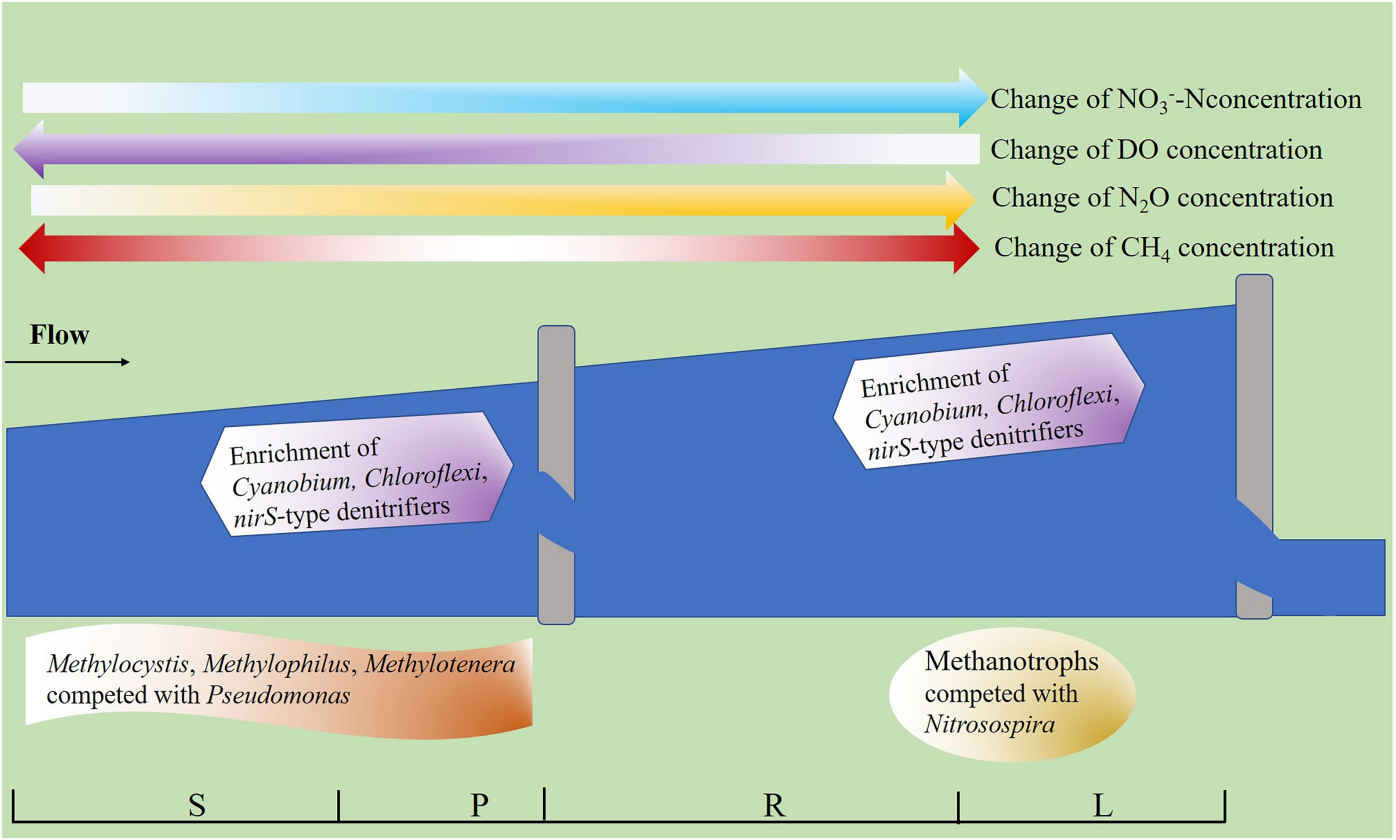
Accumulation patterns of N_2_O and CH_4_ in fragmented rivers. The water concentration of N_2_O increases with the height of barriers. The barriers (less than 2 m) inhibit CH_4_ accumulation, while barriers (higher than 2 m, less than 5 m high) promote CH_4_ accumulation. The darker the arrow, the higher the solute concentration.

In summary, we preliminarily sorted out the accumulation patterns of N_2_O and CH_4_ in fragmented rivers (Figure 9). It was found that N_2_O accumulated continuously with the increase of barriers height, and the potential reasons were related to enrichment of *Cyanobium*, *Chloroflexi*, and *nirS*-type denitrifiers, as well as the increase of NO_3_^−^-N concentration and decrease of DO concentration in water (Figure 9). The reason for the decrease of CH_4_ concentration caused by the LB may be related to the compete with *Pseudomonas* in water. The HB can lead to the accumulation of CH_4_, which was largely related to the methanotrophs to compete with *Nitrosospira* in sediment and the decrease of DO concentration.

## 5. Conclusion

In this study, the small barriers lead to the formation of different habitats, and the microbial structure and network also change. Both LB and HB reduce river velocity, leading to an increase in the water bed contact area per unit of water volume, and enrichment of *nirS*-type denitrifiers in water, promoting the accumulation of N_2_O. The LB and HB lead to the increase of water depth, which reduces the DO concentration, relieves the inhibition of oxygen on nitrate reductase activity, and promotes the accumulation of N_2_O.

In addition, the LB leads to an increase in the methanotrophs abundance in water, and an increase in the abundance of the *pmoA* gene in sediments, which reduces the accumulation of CH_4_. Moreover, the HB reduces DO concentration and *pmoA* gene abundance in water, which can increase the accumulation of CH_4_.

## Data availability statement

The datasets presented in this study can be found in online repositories. The names of the repository/repositories and accession number(s) can be found in the article/Supplementary material.

## Author contributions

C-YX, HL, QL, and ZL designed the experiments. C-YX, HL, and QL performed the experiments. C-YX and ZL analyzed the data and wrote the manuscript. L-HL was involved in interpretation of results, and figures and table arrangement. All authors revised the manuscript, read, and approved the final manuscript.

## Funding

The authors gratefully acknowledge the support from the National Key Research and Development Program of China (2022YFC3203504), the National Natural Science Foundation of China (42207243), the Chongqing Science and Technology Bureau (cstc2020jcyj18511jqX0010, sl202100000123), and China Three Gorges Corporation.

## Conflict of interest

The authors declare that the research was conducted in the absence of any commercial or financial relationships that could be construed as a potential conflict of interest.

## Publisher’s note

All claims expressed in this article are solely those of the authors and do not necessarily represent those of their affiliated organizations, or those of the publisher, the editors and the reviewers. Any product that may be evaluated in this article, or claim that may be made by its manufacturer, is not guaranteed or endorsed by the publisher.
