# Supplementary material for: Shifts in composition and function of bacterial communities reveal the effect of small barriers on nitrous oxide and methane accumulation in fragmented rivers
Source: Front Microbiol. 2023 Feb 21;14:1110025. doi: 10.3389/fmicb.2023.1110025 (PMC9990636; doi:10.3389/fmicb.2023.1110025)
Supplement: Supplementary file 1 [file Data_Sheet_1.docx]

**Shifts in the composition and function of bacterial communities** **reveal the effect of** **low barriers on nitrous oxide and methane accumulation in fragmented river**

Chong-Yang Xing^1,2^, Hang Li^1,2^, Qi Li^1,2^, Lun-Hui Lu^1,2^, Zhe Li ^1,2*^

^1^Key Laboratory of Reservoir Aquatic Environment, Chongqing Institute of Green and Intelligence Technology, Chinese Academy of Sciences, Chongqing, 400714, China

^2^ Chongqing School, University of Chinese Academy of Sciences, Chongqing, 400714, China

*Corresponding Authors

Zhe Li Email: lizhe@cigit.ac.cn

**Supplementary Material and Methods**

**1 High-throughput sequencing analysis**

Data pretreatment was based on previous literature reports (Junfeng et al. 2015). QIIME (Version 1.8.0) was applied to match filtered reads with corresponding samples (Caporaso et al. 2010). The bioinformatics analyses were all based on operational taxonomic units (OTUs), which were clustered with a 97% similarity cutoff by UPARSE (version 7.1). The chimeric sequences were identified and then removed by UCHIME (Edgar et al. 2013). The RDP Classifier against the Greengenes database was then used to obtain the taxonomy of the sequences (Liu et al. 2013). Quality controls, species annotations, and statistical calculations were carried out using the standard QIIME2 pipeline. Functional genes were predicted by using Phylogenetic Investigation of Communities by Reconstruction of Unobserved States (PICRUSt). Kyoto Encyclopedia of Genes and Genomes (KEGG) mapping was applied to characterize the metabolic pathways.

Alpha diversity indices (richness, ACE, Shannon index,Simpson index, etc.) and dissimilarity-related calculations were calculated in R Studio using the vegan and OTU table packages. Stackplot of taxonomy composition was analyzed using R studio. Bray-Curtis distance-based principal coordinate analysis (PCoA) was used to analyze the differences between samples. Analysis of similarity (ANOSIM) statistics was conducted to detect the significance of the differences between different months and sites based on Bray-Curtis dissimilarity of species using 999 permutations. PCoA, ANOSIM statistics, Wilcoxon rank-sum test, and Kruskal-Wallis H test were visualized and calculated using the Majorbio Cloud platform online (www. majorbio.com). The linear discriminant analysis effect size (LEfSe) biomarker discovery suite was to compare the abundance of the bacterial composition at each taxonomic level (from kingdom to genus) and to identify taxa differentially abundant among groups (Segata et al. 2011) between the water and sediment samples in different habitats. The relationships between the environmental factors and the alpha diversity index were analyzed by Pearson correlation analysis using R v3.6.1.

Microbial correlation network analysis is an increasingly popular tool for the study of microbial community structure and internal interactions (Rottjers et al. 2018). Two networks were constructed from water and sediment, respectively. In order to reduce noise and false positive predictions, we selected OTUs that had 100% occurrence for further analysis in each network (Liu et al. 2019). Only Spearman’s rank correlation coefficients (|r| > 0.6) and statistically significant (p < 0.05) correlations were accepted for network analyses. Topological analysis, modular analysis, and visualization were calculated by the igraph package of R Studio and Gephi (version 0.91). The size of the node was determined by the degree (the number of edges attached to the nodes) in the network.

**2 Maximum likelihood estimation method**

To further elucidate the direct and indirect effects of key explanatory variables on N_2_O concentrations and CH_4_ concentrations, we conducted path analyses using the maximum likelihood estimation method. First, a conceptual path model was developed according to existing literature and basic ecological principles. Second, promising explanatory variables were selected to include in path analysis mainly based on the results of Pearson correlation analysis. Correspondence analysis (CA) was used to reduce the variable number of microbial communities. Only the first principal component from each CA was used in the further path analyses. Third, path coefficients, R2, direct and indirect effects, and model fit parameters were calculated by AMOS 20.0 (Amos Development Corporation, Chicago, U.S.A.) software. A comparative fit index (CFI) value >0.9 and root square error of approximation (RMSEA) < 0.1 indicated that the final path model had an acceptable fit with the data.

**3 Headspace method**

Firstly, a syringe is used to extract 200 mL water sample from the water sampler. The sample should be extracted slowly during the collection process to avoid bubbles. Extract 100 mL nitrogen from an air bag containing high purity nitrogen (99.999% purity) (500 mL air bag) to form an air chamber above the syringe. Hold the syringe and shake it up and down for 3 minutes to achieve a balance between the gas-liquid phase. Push the gas in the syringe into the prepared vacuum air bag (300 mL air bag) for preservation. Record the temperature of the water after the shock. The preserved headspace samples were returned to the laboratory and the concentration of N_2_O and CH_4_ was determined by gas chromatography (Fig.S1).


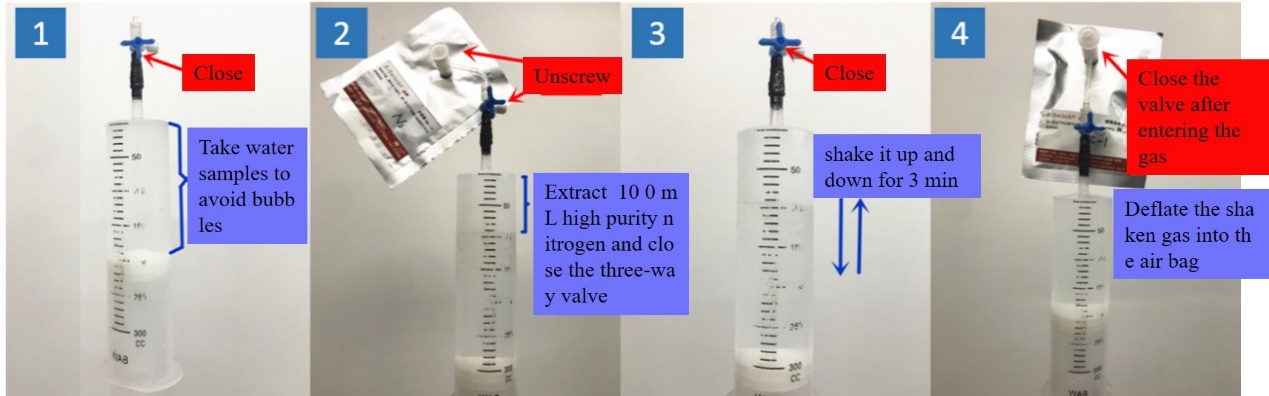


Fig. S1. Diagram of static headspace method.


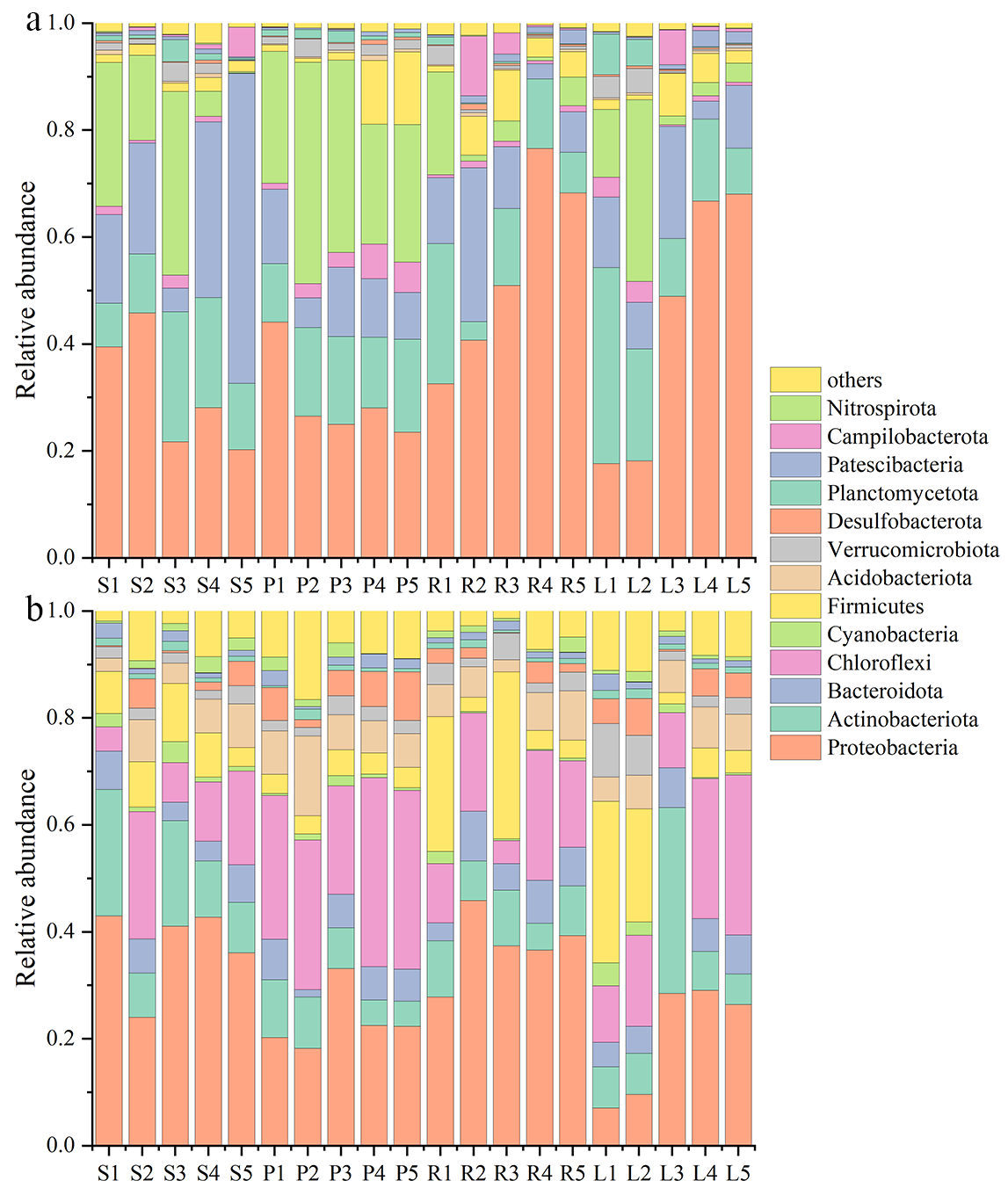


Fig. S2. Composition of microorganisms in different habitats. (a) water, (b) sediment.


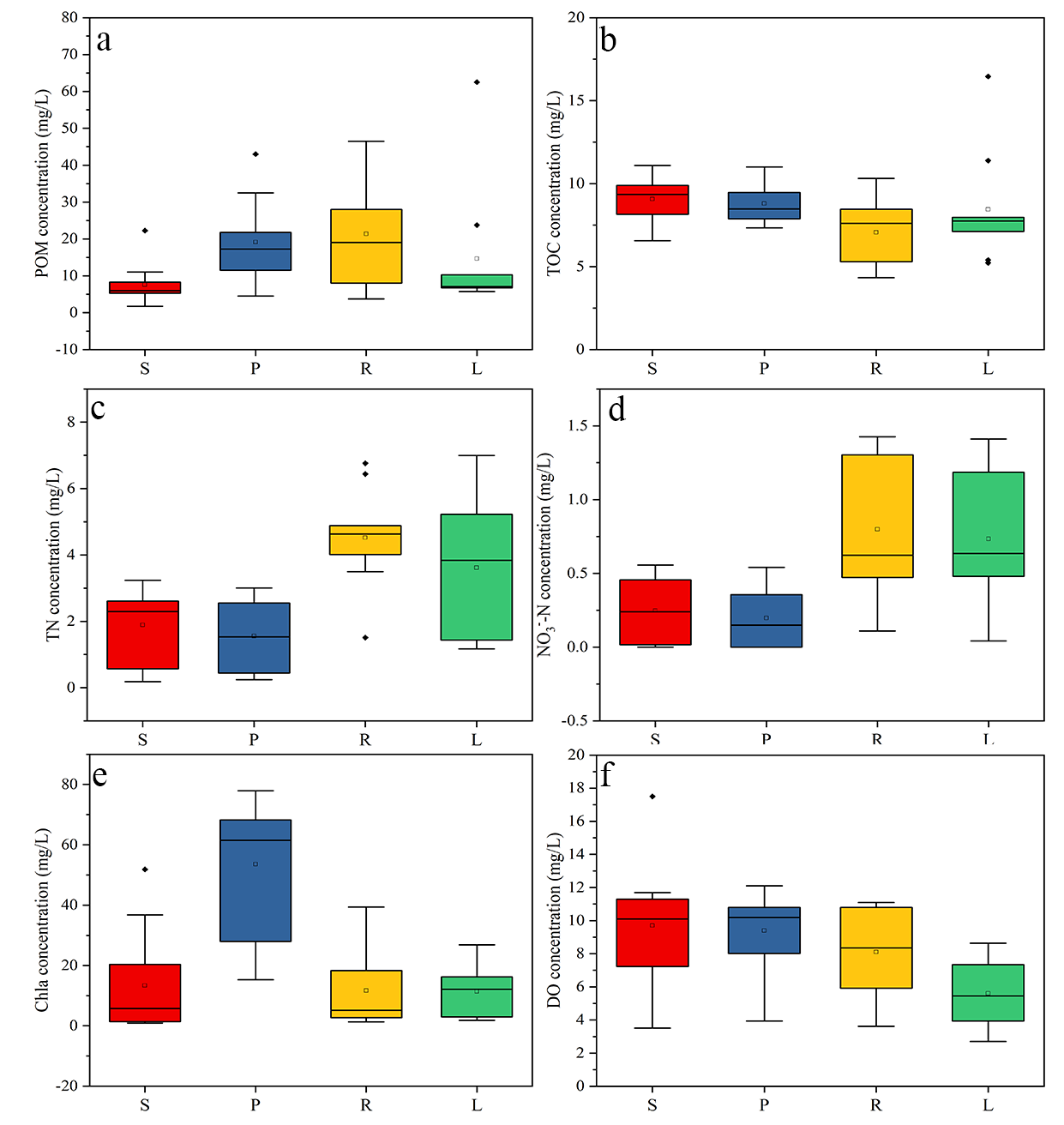


Fig. S3. Concentration of POM, TOC, TN, NO_3_^-^-N, chl *a*, and DO for the sites within different habitats.


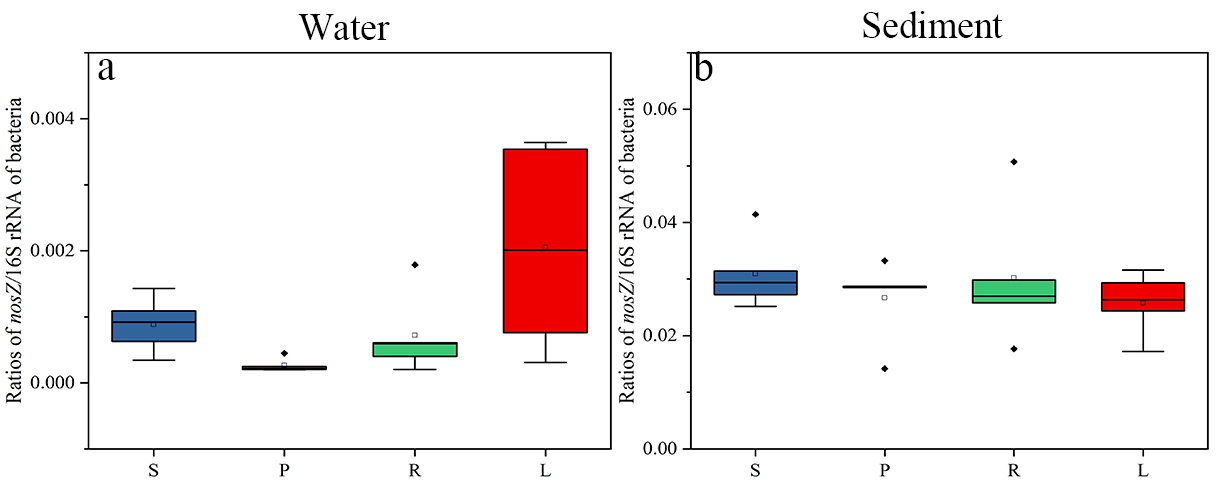


Fig. S4. Gene ratios of functional enzymes in water and sediment of different habitats. (a) *nosZ*/16S rRNA copy number ratios in water. (b) *nosZ*/16S rRNA copy number ratios in sediment.

Table S1 Characteristics of 20 sites studied in the different habitats of Liangtan River.

| Channels | Site | Latitude (N) | Longitude (E) | Max. depth (m) | Width (m) | Flow velocity (m/s) |
| --- | --- | --- | --- | --- | --- | --- |
|  | S1 | 106°19'27.1" | 29°25'54.9" | 0.10 | 1.8 | 0.14 |
|  | S2 | 106°19'28.5" | 29°25'56.8" | 0.35 | 1.6 | 0.03 |
| S | S3 | 106°20'10.6" | 29°26'42.0" | 0.20 | 1.2 | 0.01 |
|  | S4 | 106°20'19.7" | 29°27'20.8" | 0.15 | 2.2 | 0.02 |
|  | S5 | 106°19'40.1" | 29°28'33.3" | 0.19 | 2.7 | 0.03 |
|  | P1 | 106°19'26.8" | 29°25'53.7" | 0.73 | 6.8 | 0.00 |
|  | P2 | 106°19'40.3" | 29°26'12.6" | 1.00 | 8.2 | 0.00 |
| P | P3 | 106°19'39.7" | 29°28'14.8" | 0.34 | 10.3 | 0.00 |
|  | P4 | 106°21'9.79" | 29°42'19.3" | 0.50 | 7.5 | 0.00 |
|  | P5 | 106°21'10.1" | 29°42'17.3" | 0.52 | 7.8 | 0.00 |
|  | R1 | 106°19'40.6" | 29°25'13.6" | 1.50 | 13.5 | 0.04 |
|  | R2 | 106°21'52.8" | 29°38'5.68" | 2.20 | 14.3 | 0.02 |
| R | R3 | 106°21'32.1" | 29°38'39.8" | 0.32 | 11.6 | 0.03 |
|  | R4 | 106°21'31.2" | 29°41'54.8" | 2.15 | 18.7 | 0.04 |
|  | R5 | 106°22'46.5" | 29°44'59.9" | 1.25 | 12.6 | 0.03 |
|  | L1 | 106°19'22.1" | 29°25'52.6" | 4.46 | 25.6 | 0.01 |
|  | L2 | 106°20'9.42" | 29°26'40.0" | 3.49 | 33.4 | 0.00 |
| L | L3 | 106°21'32.6" | 29°38'28.8" | 2.19 | 18.6 | 0.00 |
|  | L4 | 106°21'43.3" | 29°41'55.3" | 3.06 | 20.8 | 0.00 |
|  | L5 | 106°23'47.1" | 29°45'30.0" | 4.90 | 40.1 | 0.00 |

Table S2 Primer sequences for 16S rRNA and functional genes.

| Target gene | Primer | Primer sequence（5′-3′） |
| --- | --- | --- |
| 16S rRNA | 338F  806R | ACTCCTACGGGA-GGCAGCAG  GGACTACHVGGGT-WTCTAAT |
| *nirK* | nirK876  nirK1040 | ATYGGCGGVCAYGGCGA  GCCTCGATCAGRTTRTGGTT |
| *nirS* | nirSCd3aFm  nirSR3cdm | AACGYSAAGGARACSGG  GASTTCGGRTGSGTCTTSAYGAA |
| *nosZ* | NosZ2F  NosZ2R | CGCRACGGCAASAAGGTSMSSGT  CAKRTGCAKSGCRTGGCAGAA |
| *norB* | NorB454F  NorB710R | TACTAYGARCCCTGGACTTACRA  ATGCGYGGSAWRTAGAAGWAMAMSA |
| *pmoA* | A189F | GGNGACTGGGACTTCTGG |
|  | Mb661R | CCGGMGCAACGTCYTTACC |

Table S3 Major topological properties of the empirical ecological networks of bacterial communities in water and sediment of different habitats.

|  | Habitat | Nodes | Edges | Modularity | Average  clustering  coefficient | Average  path  length | Average  degree | Linkage density | Network diameter |
| --- | --- | --- | --- | --- | --- | --- | --- | --- | --- |
| Water | S | 334 | 2809 | 1.734 | 0.411 | 3.03 | 16.82 | 0.051 | 7 |
|  | P | 287 | 2105 | 3.147 | 0.409 | 3.12 | 14.66 | 0.051 | 8 |
|  | R | 320 | 2812 | 1.969 | 0.464 | 3.19 | 17.57 | 0.055 | 9 |
|  | L | 300 | 2816 | 1.793 | 0.47 | 3.26 | 18.77 | 0.055 | 9 |
| Sediment | S | 370 | 3756 | 2.311 | 0.445 | 3.183 | 20.30 | 0.055 | 7 |
|  | P | 388 | 4789 | 1.964 | 0.492 | 3.44 | 24.68 | 0.064 | 13 |
|  | R | 409 | 4128 | 1.543 | 0.417 | 2.99 | 20.18 | 0.049 | 8 |
|  | L | 373 | 4733 | 3.181 | 0.491 | 3.12 | 25.37 | 0.068 | 12 |

Table S4 Physical and chemical surface water variables of 20 sites studied of the different habitats in winter. DO = dissolved oxygen, TP = total phosphorus, TN= total nitrogen, POM= particulate organic matter, TPM=total particulate matter, TOC = total organic carbon.

| River channels | Site | DO  (mg L^-1^) | Temperature (°C) | Conductivity (µS cm^-1^) | pH | TP  (mg L^-1^) | TN  (mg L^-1^) | NO_3_^-^-N  (mg L^-1^) | NO_2_^-^-N  (mg L^-1^) | NH_4_^+^-N  (mg L^-1^) | chl *a*  (µg L^-1^) | POM  (mg L^-1^) | TPM  (mg L^-1^) | TOC  (mg L^-1^) |
| --- | --- | --- | --- | --- | --- | --- | --- | --- | --- | --- | --- | --- | --- | --- |
|  | S1 | 11.30 | 12.10 | 402.00 | 7.43 | 0.460 | 2.275 | 0.382 | 0.005 | 1.883 | 51.846 | 22.25 | 92.00 | 10.37 |
|  | S2 | 10.70 | 11.4 | 418.00 | 7.68 | 0.216 | 2.595 | 0.411 | 0.019 | 1.920 | 20.370 | 8.25 | 28.50 | 11.09 |
| S | S3 | 11.70 | 9.30 | 359.00 | 8.60 | 0.041 | 2.612 | 0.456 | 0.004 | 1.385 | 8.085 | 1.75 | 4.75 | 9.90 |
|  | S4 | 9.70 | 8.70 | 399.00 | 8.80 | 0.146 | 2.847 | 0.530 | 0.012 | 1.658 | 4.766 | 6.25 | 24.00 | 8.42 |
|  | S5 | 10.50 | 8.30 | 398.00 | 7.01 | 0.182 | 3.235 | 0.557 | 0.024 | 0.086 | 1.668 | 2.25 | 6.50 | 9.31 |
|  | P1 | 8.01 | 8.70 | 395.00 | 8.09 | 0.139 | 2.401 | 0.356 | 0.010 | 1.540 | 60.315 | 11.50 | 21.75 | 8.92 |
|  | P2 | 12.10 | 8.80 | 388.00 | 8.60 | 0.132 | 3.004 | 0.449 | 0.013 | 1.618 | 57.910 | 15.25 | 21.25 | 10.60 |
| P | P3 | 10.80 | 8.80 | 366.00 | 7.88 | 0.142 | 2.789 | 0.541 | 0.030 | 0.129 | 15.253 | 4.50 | 7.00 | 7.87 |
|  | P4 | 10.30 | 9.13 | 446.00 | 7.80 | 0.160 | 2.551 | 0.325 | 0.004 | 0.262 | 68.252 | 16.50 | 32.50 | 9.47 |
|  | P5 | 10.00 | 9.15 | 450.00 | 7.85 | 0.181 | 2.285 | 0.285 | 0.005 | 1.229 | 77.925 | 18.00 | 43.00 | 11.01 |
|  | R1 | 11.10 | 11.70 | 416.00 | 7.60 | 0.181 | 3.500 | 0.440 | 0.015 | 0.471 | 28.355 | 11.00 | 46.50 | 10.31 |
|  | R2 | 8.71 | 12.10 | 923.00 | 7.77 | 0.332 | 4.048 | 1.426 | 0.190 | 0.094 | 3.092 | 5.25 | 24.50 | 7.30 |
| R | R3 | 10.80 | 11.60 | 842.00 | 7.82 | 0.506 | 6.440 | 1.033 | 0.239 | 0.088 | 3.319 | 7.50 | 44.50 | 8.60 |
|  | R4 | 5.23 | 11.60 | 836.00 | 7.66 | 0.469 | 6.763 | 1.304 | 0.178 | 0.155 | 1.995 | 5.25 | 22.50 | 8.45 |
|  | R5 | 9.19 | 10.60 | 889.00 | 7.64 | 0.453 | 4.763 | 1.417 | 0.189 | 0.416 | 2.729 | 5.75 | 28.00 | 8.11 |
|  | L1 | 4.50 | 10.17 | 362.00 | 7.64 | 0.144 | 3.423 | 0.661 | 0.012 | 0.074 | 14.032 | 6.75 | 8.25 | 11.39 |
|  | L2 | 2.70 | 9.70 | 355.00 | 8.40 | 0.062 | 1.918 | 0.350 | 0.020 | 0.151 | 22.493 | 6.75 | 8.50 | 16.45 |
| L | L3 | 8.25 | 11.40 | 857.00 | 7.66 | 0.412 | 6.993 | 1.185 | 0.200 | 0.054 | 1.837 | 23.75 | 20.75 | 7.96 |
|  | L4 | 5.45 | 11.60 | 837.00 | 7.69 | 0.504 | 5.361 | 1.297 | 0.178 | 0.830 | 2.155 | 7.00 | 19.25 | 7.94 |
|  | L5 | 8.64 | 10.40 | 890.00 | 7.67 | 0.430 | 5.146 | 1.411 | 0.182 | 0.217 | 2.931 | 7.00 | 20.25 | 7.72 |

Table S5 Physical and chemical surface water variables of 20 sites studied of the different habitats in summer. DO = dissolved oxygen, TP = total phosphorus, TN= total nitrogen, POM= particulate organic matter, TPM=total particulate matter, TOC = total organic carbon.

| River channels | Site | DO  (mg L^-1^) | Temperature (°C) | Conductivity (µS cm^-1^) | pH | TP  (mg L^-1^) | TN  (mg L^-1^) | NO_3_^-^-N  (mg L^-1^) | NO_2_^-^-N  (mg L^-1^) | NH_4_^+^-N  (mg L^-1^) | chl *a*  (µg L^-1^) | POM  (mg L^-1^) | TPM  (mg L^-1^) | TOC  (mg L^-1^) |
| --- | --- | --- | --- | --- | --- | --- | --- | --- | --- | --- | --- | --- | --- | --- |
|  | S1 | 9.23 | 30.50 | 302.00 | 7.43 | 0.057 | 0.513 | 0.017 | 0 | 0.096 | 36.750 | 6.00 | 76.00 | 9.40 |
|  | S2 | 7.23 | 31.40 | 318.00 | 7.37 | 0.276 | 2.306 | 0.097 | 0 | 0.221 | 6.775 | 10.50 | 76.25 | 9.44 |
| S | S3 | 17.50 | 32.80 | 308.90 | 8.82 | 0.081 | 0.182 | 0.019 | 0 | 0.479 | 1.417 | 3.00 | 40.50 | 8.15 |
|  | S4 | 3.51 | 26.00 | 432.20 | 7.19 | 0.171 | 0.572 | 0 | 0.001 | 0.550 | 0.925 | 3.00 | 52.00 | 8.09 |
|  | S5 | 5.71 | 29.23 | 437.80 | 7.37 | 0.356 | 1.773 | 0 | 0.076 | 0.134 | 0.875 | 6.00 | 70.00 | 6.56 |
|  | P1 | 3.94 | 28.00 | 378.40 | 7.19 | 0.085 | 0.678 | 0 | 0 | 0.199 | 62.683 | 14.00 | 53.75 | 8.65 |
|  | P2 | 11.67 | 28.60 | 322.70 | 8.51 | 0.203 | 0.443 | 0 | 0 | 0.237 | 28.000 | 15.75 | 83.75 | 8.26 |
| P | P3 | 6.44 | 30.50 | 434.80 | 7.37 | 0.109 | 0.406 | 0 | 0 | 0.164 | 24.975 | 10.25 | 62.25 | 7.52 |
|  | P4 | 10.09 | 32.70 | 454.70 | 7.78 | 0.141 | 0.780 | 0.13 | 0 | 0.217 | 73.125 | 15.75 | 67.50 | 8.29 |
|  | P5 | 10.65 | 32.30 | 450.00 | 7.86 | 0.147 | 0.246 | 0.00 | 0 | 0.223 | 67.150 | 17.00 | 85.50 | 7.33 |
|  | R1 | 7.61 | 29.60 | 363.00 | 7.73 | 0.132 | 1.511 | 0.109 | 0 | 0.910 | 39.375 | 8.00 | 46.75 | 7.90 |
|  | R2 | 5.92 | 28.20 | 630.60 | 7.34 | 0.371 | 4.778 | 0.646 | 0.070 | 0.299 | 1.325 | 15.50 | 70.00 | 5.69 |
| R | R3 | 7.99 | 29.10 | 681.00 | 7.87 | 0.371 | 4.885 | 0.543 | 0.118 | 0.221 | 6.950 | 8.00 | 82.25 | 5.30 |
|  | R4 | 3.62 | 27.90 | 579.90 | 7.35 | 0.725 | 4.500 | 0.472 | 0.035 | 0.771 | 11.100 | 12.50 | 199.50 | 4.34 |
|  | R5 | 10.88 | 36.10 | 456.00 | 8.40 | 0.393 | 4.015 | 0.599 | 0.081 | 0.378 | 18.344 | 3.75 | 65.00 | 4.57 |
|  | L1 | 2.93 | 27.70 | 320.50 | 8.51 | 0.082 | 1.169 | 0 | 0.006 | 0.506 | 26.875 | 9.50 | 51.00 | 7.11 |
|  | L2 | 3.94 | 28.30 | 331.00 | 8.63 | 0.127 | 1.228 | 0.042 | 0.000 | 0.221 | 11.500 | 5.75 | 19.75 | 7.78 |
| L | L3 | 5.45 | 29.80 | 644.80 | 7.46 | 0.250 | 5.221 | 0.636 | 0.038 | 0.442 | 3.050 | 7.25 | 59.25 | 5.22 |
|  | L4 | 7.34 | 31.30 | 635.00 | 7.71 | 2.397 | 1.436 | 0.480 | 0.011 | 0.397 | 12.675 | 62.50 | 206.75 | 7.47 |
|  | L5 | 6.85 | 30.40 | 533.20 | 7.62 | 0.351 | 4.255 | 0.535 | 0.051 | 0.463 | 16.225 | 10.25 | 80.25 | 5.39 |

| River channels | Sampling site | *nirS*  copies mL^-1^ | *nirK*  copies mL^-1^ | *nosZ*  copies mL^-1^ | *pmoA*  copies mL^-1^ | | 16SrRNA copies mL^-1^ | |
| --- | --- | --- | --- | --- | --- | --- | --- | --- |
| S | S1 | 177.9 | 218.5 | 2.1 | 154.2 | 1925.2 | |  |
|  | S2 | 28.1 | 77.3 | 0.4 | 57.6 | 1163.5 | |  |
|  | S3 | 2.4 | 20.8 | 0.05 | 14.8 | 79.7 | |  |
|  | S4 | 33.8 | 98.7 | 0.4 | 27.1 | 433.8 | |  |
|  | S5 | 29.4 | 43.5 | 1.5 | 34.7 | 1048.2 | |  |
| P | P1 | 22.9 | 35.2 | 0.3 | 77.5 | 669.0 | |  |
|  | P2 | 9.8 | 53.0 | 0.1 | 7.3 | 494.5 | |  |
|  | P3 | 6.1 | 13.4 | 0.05 | 8.7 | 200.7 | |  |
|  | P4 | 102.8 | 81.2 | 0.3 | 132.3 | 1363.3 | |  |
|  | P5 | 112.5 | 165.7 | 0.4 | 131.0 | 1939.4 | |  |
| R | R1 | 30.7 | 194.9 | 0.9 | 51.1 | 4383.0 | |  |
|  | R2 | 36.6 | 75.0 | 1.2 | 3.8 | 671.2 | |  |
|  | R3 | 24.1 | 112.5 | 0.6 | 4.0 | 986.0 | |  |
|  | R4 | 85.6 | 185.7 | 1.5 | 6.4 | 3728.0 | |  |
|  | R5 | 44.1 | 46.9 | 0.5 | 3.2 | 842.5 | |  |
| L | L1 | 3.9 | 40.8 | 0.3 | 32.9 | 394.6 | |  |
|  | L2 | 13.9 | 66.0 | 0.3 | 57.6 | 966.9 | |  |
|  | L3 | 49.5 | 45.0 | 3.1 | 6.0 | 876.1 | |  |
|  | L4 | 56.0 | 38.0 | 1.9 | 3.5 | 521.7 | |  |
|  | L5 | 68.2 | 32.4 | 1.3 | 5.2 | 646.8 | |  |

Table S6 Average copy number (×10^3^) of functional enzyme genes (*nirS*, *nirK*, *nosZ* and *pmoA* ) and 16S rRNA from water under different habitats in winter.

| River channels | Sampling site | *nirS*  copies mL^-1^ | *nirK*  copies mL^-1^ | *NosZ*  copies mL^-1^ | *pmoA*  copies mL^-1^ | | 16SrRNA copies mL^-1^ | |
| --- | --- | --- | --- | --- | --- | --- | --- | --- |
| S | S1 | 6.0 | 239.7 | 10.3 | 110.1 | 722.9 | |  |
|  | S2 | 40.5 | 480.8 | 17.5 | 80.8 | 679.4 | |  |
|  | S3 | 0.5 | 8.77 | 1.7 | 4.6 | 17.9 | |  |
|  | S4 | 29.5 | 805.1 | 90.7 | 213.2 | 1397.5 | |  |
|  | S5 | 3.03 | 84.3 | 16.0 | 16.4 | 155.0 | |  |
| P | P1 | 3.5 | 107.8 | 10.7 | 128.0 | 524.3 | |  |
|  | P2 | 37.8 | 1036.8 | 41.0 | 123.8 | 2169.0 | |  |
|  | P3 | 5.8 | 545.5 | 30.4 | 151.5 | 1582.0 | |  |
|  | P4 | 16.4 | 480.4 | 37.4 | 136.3 | 755.1 | |  |
|  | P5 | 33.2 | 1244.1 | 64.4 | 171.2 | 1267.6 | |  |
| R | R1 | 22.5 | 700.6 | 30.4 | 216.9 | 843.8 | |  |
|  | R2 | 2.87 | 45.4 | 9.9 | 1.9 | 77.0 | |  |
|  | R3 | 5.77 | 69.7 | 9.9 | 46.8 | 137.1 | |  |
|  | R4 | 225.4 | 460.8 | 131.9 | 224.9 | 1163.3 | |  |
|  | R5 | 35.4 | 140.7 | 24.5 | 137.5 | 522.3 | |  |
| L | L1 | 35.5 | 280.1 | 31.5 | 20.2 | 3943.5 | |  |
|  | L2 | 32.0 | 827.5 | 64.5 | 1294 | 2691.9 | |  |
|  | L3 | 27.4 | 128.3 | 52.4 | 77.1 | 725.7 | |  |
|  | L4 | 320.5 | 570.1 | 159.7 | 230.9 | 1897.5 | |  |
|  | L5 | 36.5 | 288.7 | 37.3 | 96.1 | 1027.4 | |  |

Table S7 Average copy number (×10^3^) of functional enzyme genes (*nirS*, *nirK*, *nosZ* and *pmoA*) and 16S rRNA from water under different habitats in summer.

Table S8 Average copy number (×10^6^) of functional enzyme genes (*nirS*, *nirK*, *nosZ* and *pmoA* ) and 16S rRNA from sediment under different habitats in winter.

| Habitat conditions | Sampling site | *nirS*  copies g^-1^ | *nirK*  copies g^-1^ | *nosZ* copies g^-1^ | *pmoA*  copies g^-1^ | 16S rRNA copies g^-1^ |
| --- | --- | --- | --- | --- | --- | --- |
| S | S1 | 232.9 | 1219.5 | 108.7 | 48.4 | 3457.6 |
|  | S2 | 176.4 | 1537.2 | 74.9 | 43.1 | 2975.8 |
|  | S3 | 129.5 | 702.6 | 71.6 | 10.4 | 2438.0 |
|  | S4 | 7.0 | 78.7 | 5.5 | 1.25 | 202.0 |
|  | S5 | 1.0 | 411.3 | 38.7 | 21.4 | 934.0 |
| P | P1 | 86.2 | 436.9 | 17.9 | 11.4 | 1262.2 |
|  | P2 | 12.7 | 257.7 | 15.7 | 1.10 | 472.0 |
|  | P3 | 295.5 | 1458.7 | 108.7 | 45.6 | 3780 |
|  | P4 | 220.2 | 772.1 | 80.3 | 16.6 | 2817.6 |
|  | P5 | 210.3 | 990.1 | 87.9 | 7.6 | 3058.5 |
| R | R1 | 255.3 | 1595.9 | 69.9 | 50.1 | 3953.3 |
|  | R2 | 188.7 | 1054.8 | 36.3 | 7.5 | 715.6 |
|  | R3 | 187.5 | 1087.6 | 18.6 | 0.91 | 689.7 |
|  | R4 | 499.2 | 1120.4 | 35.9 | 27.3 | 1390.8 |
|  | R5 | 324.8 | 1153.1 | 35.0 | 9.5 | 1171.6 |
| L | L1 | 43.4 | 771.2 | 66.7 | 31.1 | 3872.7 |
|  | L2 | 43.6 | 317 | 51.4 | 13.2 | 2108.8 |
|  | L3 | 36.5 | 451.8 | 48.3 | 1.8 | 1528.3 |
|  | L4 | 201.3 | 463.3 | 62.3 | 16.3 | 2362.4 |
|  | L5 | 156.0 | 780.8 | 52.3 | 13.3 | 1782.0 |

| Habitat conditions | Sampling site | *nirS*  copies g^-1^ | *nirK*  copies g^-1^ | *nosZ* copies g^-1^ | *pmoA*  copies g^-1^ | 16S rRNA copies g^-1^ |
| --- | --- | --- | --- | --- | --- | --- |
| S | S1 | 557.0 | 1728.1 | 131.9 | 120.6 | 2552.7 |
|  | S2 | 154.5 | 5.3 | 24.7 | 65.2 | 365.7 |
|  | S3 | 1043.5 | 1486.4 | 156.3 | 176.4 | 2686.8 |
|  | S4 | 28.2 | 23.6 | 11.9 | 537.3 | 155.8 |
|  | S5 | 355.3 | 174.6 | 65.5 | 60.7 | 699.0 |
| P | P1 | 227.6 | 320.2 | 76.2 | 136.1 | 687.1 |
|  | P2 | 113.9 | 247.1 | 44.1 | 49.9 | 404.9 |
|  | P3 | 452.2 | 640.4 | 148.6 | 208.0 | 1462.2 |
|  | P4 | 462.7 | 1179.3 | 292.8 | 36.8 | 2130.1 |
|  | P5 | 147.3 | 339.6 | 89.5 | 17.3 | 713.2 |
| R | R1 | 124.4 | 100.5 | 15.3 | 19.1 | 204.1 |
|  | R2 | 277.3 | 180.0 | 36.5 | 22.1 | 688.3 |
|  | R3 | 22.2 | 33.2 | 7.5 | 2.1 | 98.3 |
|  | R4 | 17.0 | 37.6 | 9.6 | 3.7 | 60.4 |
|  | R5 | 83.4 | 706.9 | 94.3 | 8.2 | 1209.0 |
| L | L1 | 58.7 | 158.5 | 27.7 | 33.8 | 880.3 |
|  | L2 | 61.7 | 143.0 | 23.7 | 43.7 | 706.1 |
|  | L3 | 0 | 0 | 0 | 0 | 0.8 |
|  | L4 | 68.5 | 74.1 | 18.8 | 10.9 | 257.3 |
|  | L5 | 168.4 | 149.1 | 30.9 | 21.7 | 641.7 |

Table S9 Average copy number (×10^6^) of functional enzyme genes (*nirS*, *nirK*, *nosZ* and *pmoA* ) and 16S rRNA from sediment under different habitats in summer.

**References**

Caporaso, J. G. Caporaso, J.G., Kuczynski, J., Stombaugh, J., Bittinger, K., Bushman, F.D., Costello, E.K., Fierer, N., Pena, A.G., Goodrich, J.K., Gordon, J.I., Huttley, G.A., Kelley, S.T., Knights, D., Koenig, J.E., Ley, R.E., Lozupone, C.A., McDonald, D., Muegge, B.D., Pirrung, M., Reeder, J., Sevinsky, J.R., Tumbaugh, P.J., Walters, W.A., Widmann, J., Yatsunenko, T., Zaneveld, J. and Knight, R*.* 2010. QIIME allows analysis of high-throughput community sequencing data. Nat Methods. 7, 335–336.

Edgar, R. C. 2013. UPARSE: highly accurate OTU sequences from microbial amplicon reads. Nat Methods. 10, 996–999.

Junfeng, L. Junfeng, L., Junyi, Z., Liyang, L., Yucai, F., Lianshuo, L., Yunfeng, Y., Zuhong, L. and Xuegong, Z. 2015. Annual periodicity in planktonic bacterial and archaeal community composition of eutrophic Lake Taihu. Sci rep. 5.

Liu, C., Hou, L., Liu, M., Zheng, Y., Yin, G., Han, P., Dong, H., Gao, J., Gao, D., Chang, Y. and Zhang, Z.. 2019. Response of the eukaryotic plankton community to the cyanobacterial biomass cycle over 6 years in two subtropical reservoirs. ISME J. 13, 2196–2208.

Liu KL, and Wong TT. 2013. Naive bayesian classifiers with multinomial models for rRNA taxonomic assignment. IEEE-ACM Trans Comput Biol Bioinform. 10, 1334–1339.

Rottjers, L. and Faust, K. 2018. From hairballs to hypotheses-biological insights from microbial networks. FEMS Microbiol Rev. 42, 761–780.

Segata, N. Segata, N., Izard, J., Waldron, L., Gevers, D., Miropolsky, L., Garrett, W.S. and Huttenhower, C*.* 2011. Metagenomic biomarker discovery and explanation. Genome Biol. 12.
